# One-Pot Synthesis of 2,3,4-Triarylquinolines via Suzuki-Miyaura Cross-Coupling of 2-Aryl-4-chloro-3-iodoquinolines with Arylboronic Acids

**DOI:** 10.3390/molecules15107423

**Published:** 2010-10-22

**Authors:** Malose Jack Mphahlele, Mamasegare Mabel Mphahlele

**Affiliations:** Department of Chemistry, College of Science, Engineering and Technology, University of South Africa, P.O. Box 392, Pretoria 0003, South Africa

**Keywords:** 2-aryl-4-chloro-3-iodoquinolines, Suzuki-Miyaura cross-coupling, 2,3-diaryl-4-chloroquinolines, 2,3,4-triarylquinolines

## Abstract

Palladium–catalyzed Suzuki cross-coupling of 2-aryl-4-chloro-3-iodoquinolines with excess arylboronic acids (2.5 equiv.) in the presence of tricyclohexylphosphine afforded the 2,3,4-triarylquinolines in one-pot operation. The incipient 2,3-diaryl-4-chloroquinolines were also prepared and transformed to the primary 4-amino-2,3-diarylquinolines and 2,3-diarylquinolin-4(1*H*)-ones.

## 1. Introduction

The high reactivity of the aryl-iodo bond toward oxidative addition with palladium in Suzuki [1,2,3,4], Sonogashira [4,5], Stille [4] and Heck [4] cross-coupling reactions has been found to allow successive substitution of the halogen atoms (I>Br >Cl>>F) in dihaloquinolines. The observed trend relates to the Ar–X bond strength, which increases as follows: I<Br<Cl<F (*D*_Ph-X_ values 65, 81, 96 and 126 Kcal/mol, respectively) and makes the oxidative addition step increasingly difficult [6]. We have previously subjected a series of 2-aryl-4-chloro-3-iodoquinolines to Suzuki cross-coupling with phenylboronic acid (1.2–2.0 equiv.) using tetrakis(triphenylphosphine)palladium(0) (Pd(PPh_3_)_4_) as catalyst and 2M K_2_CO_3_ in dimethyl formamide (DMF) under reflux to afford the 2,3-diaryl-4-chloroquinolines in moderate yields [1]. Hitherto our investigation, the analogous 4-chloro-6-(bromo/iodo)quinolines were subjected to successive replacement of the two halogen atoms via Suzuki cross-coupling to afford the C*sp*^2^–C*sp*^2^ cross-coupled products [2,3]. The second arylboronic acid was in this case added to the reaction mixture after completion of the first step (tlc monitoring) without isolating the incipient 6-substituted derivative. Despite the successes in sequential metal-catalyzed halogen substitution reactions [2,3,4], the development of versatile and efficient methods for the synthesis of polysubstituted quinolines from dihaloquinolines in a single operation remains a challenge in organic synthesis. We are interested in the synthesis of 3,4-disubstituted 2-arylquinoline derivatives as a prelude to derivatives with potential biological activity or photoelectronic properties and the 2-aryl-4-chloro-3-iodoquinolines appeared suitable candidates for palladium-catalyzed Suzuki cross-coupling to afford such systems. 

As we have previously communicated, Suzuki cross-coupling of the 2-aryl-4-chloro-3-iodoquinolines with phenylboronic acid did not proceed beyond C-3 substitution after 48 hours [1]. The slow oxidative addition step using Pd(0)(PPh_3_)_4_ as a precursor of palladium(0) complex is attributed to the inhibiting role of the extra PPh_3_ generated in the 2^nd^ equilibrium {*S*Pd(0)(PPh_3_)_3_⇌*S*Pd(0)(PPh_3_)_2_ + PPh_3_ (*K*_2_/[PPh_3_] << 1); *S* = solvent} to afford the reactive low ligated 14-electron species (Pd(0)(PPh_3_)_2_) [7]. The oxidative addition performed from palladium(0) complex (Pd(0)(PPh_3_)_2_Cl^¯^) generated by the reduction of dichlorobis(triphenylphosphine)palladium(II) (PdCl_2_(PPh_3_)_2_) is reported to be more than 30 times faster than that performed from Pd(0)(PPh_3_)_4_ [7]. Likewise, alkylphosphine ligands are known to coordinate with palladium and increase its electron density than arylphosphines and, in turn, accelerate the oxidative addition and reductive elimination steps in the catalytic cycle [8,9]. Based on this postulate we decided to investigate the possibility for the direct one-pot synthesis of 2,3,4-triarylquinolines via palladium-catalyzed Suzuki-Miyaura cross-coupling of 2-aryl-4-chloro-3-iodoquinolines with arylboronic acids as models for C–C bond formation.

## 2. Results and Discussion 

**Scheme 1:**
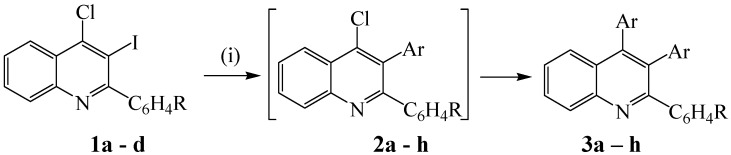
Suzuki-Miyaura cross-coupling of 2-aryl-4-chloro-3-iodoquinolines.

We subjected the known 2-aryl-4-chloro-3-iodoquinolines **1** [1] to PdCl_2_(PPh_3_)_2_–catalyzed Suzuki cross-coupling with arylboronic acid derivatives (2.5 equiv.) in the presence of tricyclohexylphosphine (PCy_3_) and K_2_CO_3_ in dioxane-water (3:1, v/v) (Scheme 1). The reaction in the presence of PdCl_2_(PPh_3_)_2_–PCy_3_ catalyst mixture was complete within 18 hours without any trace of the starting material. We isolated in all cases by column chromatography a single product characterized using a combination of spectroscopic techniques(NMR, IR, MS) as the corresponding 2,3,4-triarylquinoline **3**. In some cases, the 2,3-diaryl-4-chloroquinoline **2** was detected in the reaction mixture by thin layer chromatography, but could not be isolated by column chromatography. The 2,3-diarylquinolines substituted at the C-4 position with H, CH_3_, NH_2_, CO_2_H or Ph have been found to serve as selective cyclooxygenase-1/-2 (COX-1 or COX-2) inhibitors [10]. 2-Arylquinolines bearing vinyl, alkynyl, halogen (Cl, Br) or phenyl substituent on the C-4 position, on the other hand, were found to display high affinity (3–5 nM) and significant selectivity (up to 83-fold) for estrogen receptor β (ERβ) [11]. Moreover, the analogous 2,4-diarylquinolines show intense blue emission upon UV excitation [12].

**Figure 1:**
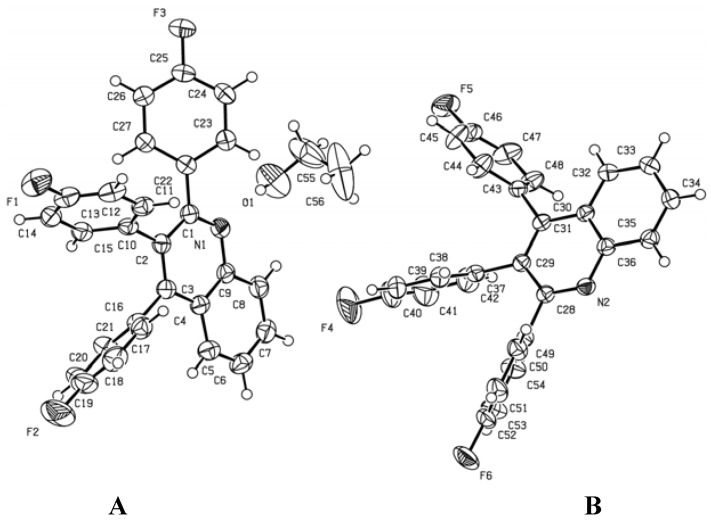
X-ray crystal structure of 2,3,4-tris(4-fluorophenyl)quinoline **3f** showing crystallographic numbering. For clarity, hydrogen atoms are not labelled.

Crystals of quality suitable for X-ray diffraction were obtained for **3f** and the molecular structure of these novel systems were further confirmed by X-ray diffraction. Compound **3f** crystallizes in the triclinic space group *P-*1 [a = 10.2571(2), b = 13.2887(2), c = 16.7681(3) Å; α = 103.289(1)°, β = 99.454(1)°, γ = 96.939(1)°] with two independent molecules (**A** and **B**) and an ethanol molecule in the asymmetric unit (Fig. 1). One of the molecules (**A**) is hydrogen bonded to ethanol: O(1)-H(1) 0.84 Å; H(1)^…^N(1) 2.11 Å; O(1)^…^N(1) 2.919(2) Å; <O(1)H(1)N(1) 161°. The 2-, 3- and 4-aryl rings of both molecules in the unit are strongly deformed out of plane of the quinoline ring as evidenced by the large torsion angles (Table 1) [13]. The 2-aryl substituent of molecule (**A**) is however relatively less deformed (N(1)-C(1)-C(22)-C(23) = 42.09°) due to the hydrogen bonded ethanol molecule. Crystal data and experimental details for compound **3f** are shown in Table 2.

**Scheme 2:**
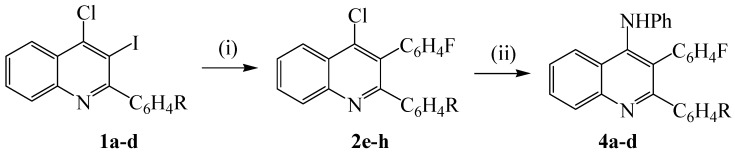
Successive C-3 arylation and amination of **1**.

Since the 2-aryl-4-chloro-3-(4-fluorophenyl)quinolines **2e**-**h** have not been described before and were in some cases only detected in the reaction mixtures, we decided to prepare these systems from **1**. We followed a similar procedure previously employed for the synthesis of **2a**-**d** [1] and subjected systems **1** to 4-fluorophenylboronic acid (1.2 equiv.) in the presence of Pd(0)(PPh_3_)_4_ and 2M K_2_CO_3_ as a base in DMF. We isolated in all cases the corresponding 3-(4-fluorophenyl) derivatives **2e-h** as sole products (Scheme 2). The presence of a fluorine atom in quinolones and quinoline derivatives is known to have a profound effect on their biological, chemical and physical properties [1,14,15]. With this consideration in mind, we took advantage of the known ease of displacement of the 4-chloro atom on the quinoline ring by nucleophiles and subjected systems **2e-h** to aniline in dioxane under reflux (Scheme 2). We isolated the corresponding primary 4-amino 2,3-diarylquinolines **4** with potential antimalarial [16-18], anti-inflammatory [19], and antihypertensive activities [20]. The primary 4-amino-2-arylquinolines also represent a novel class of NR1/2B subtype selective *N*-methyl-D-aspartate (NMDA) receptor antagonists [21].

**Scheme 3:**
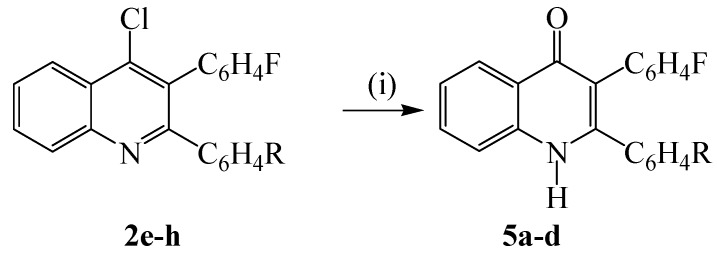
Hydrolysis of **2** to NH-4-oxo derivatives **5**.

To further demonstrate the versatility of the 4-chloroquinoline derivatives in synthesis in the last part of this investigation, we decided to investigate the possibility of transforming systems **2e-f** to the NH-4-oxo derivatives. Whereas the NMe-4-oxo [22] or NPh-4-oxo [23] derivatives undergo Suzuki cross-coupling with arylboronic acids with ease to afford the corresponding *N*-substituted 2,3-diarylquinolinones, under similar reaction conditions the NH-4-oxo precursors afford complex mixtures of products [22]. Although demethylation of 2,3-diaryl-4-methoxyquinolines with boron tribromide in dichloromethane afforded the 2,3-diarylquinolin-4(1*H*)-ones, under these reaction conditions the 4-methoxy-2-(4-methoxyphenyl)-3-phenylquinoline led to a complex mixture of products lacking the methoxy signals in the ^1^H-NMR spectrum [1]. Consequently, in this investigation we subjected systems **2e**-**h** to acetic acid/water (4:1, v/v) under reflux and we isolated the corresponding previously undescribed 2-aryl-3-(4-fluorophenyl)quinolin-4(1*H*)-ones **5a-d** in high yield and purity (Scheme 3). The smooth hydrolysis of the 4-chloroquinolines to afford the NH-4-oxo derivatives without affecting the 4-methoxy group make this a convenient synthetic strategy for the construction of 2,3-diarylquinolin-4(1*H*)-ones that are difficult to synthesize otherwise. 

## 3. Experimental

### 3.1. General

Melting points were recorded on a Thermocouple digital melting point apparatus. IR spectra were recorded as powders using FTS 7000 Series Digilab Win-IR Pro ATR (attenuated total reflectance) spectrometer. For column chromatography, Merck Kieselgel 60 (0.063–0.200 mm) was used as stationary phase. NMR spectra were obtained using a Varian Mercury 300 MHz NMR spectrometer and the chemical shifts are measured relative to the solvent peaks. Low and high-resolution mass spectra were recorded at an ionization potential of 70eV using a Micromass Autospec-TOF (double focusing high resolution) instrument. The synthesis and characterization of substrates **1** have been described before [1].

### 3.2. Typical procedure for the one-pot synthesis of 2,3,4-triarylquinolines **2**

2-Aryl-4-chloro-3-iodoquinoline **1** (1 equiv.), arylboronic acid (2.5 equiv.), PdCl_2_(PPh_3_)_2_ (5% of **1**), PCy_3_ (10% of **1**), K_2_CO_3_ (2 equiv.) and 3:1 dioxane–water (*ca*. 5 mL/mmol of **1**) were added to a two-necked flask equipped with a stirrer bar, rubber septum and a condenser. The mixture was flushed for 20 minutes with argon gas and a balloon filled with argon gas was connected to the top of the condenser. The mixture was heated with stirring at 80–90 °C under argon atmosphere for 18 hours and then allowed to cool to room temperature. The cooled mixture was poured into ice-cold water and the product was taken-up into chloroform. The combined organic extracts were washed with brine, dried over anhydrous MgSO_4_, filtered and then evaporated under reduced pressure. The residue was purified by column chromatography to afford the 2,3,4-triarylquinoline **3**. The following products were prepared in this fashion:

2,*3,4-Triphenylquinoline* (**3a**). A mixture of **1a** (0.50 g, 1.37 mmol), phenylboronic acid (0.42 g, 3.42 mmol), PdCl_2_(PPh_3_)_2_ (0.05 g, 0.07 mmol), PCy_3_ (0.04 g, 0.14 mmol), and K_2_CO_3_ (0.38 g, 2.74 mmol) in dioxane/water (20 mL) afforded (**3a**) as a solid (0.29 g, 59%), mp 197–198 °C (ethanol); *R_f_* (10% ethyl acetate/hexane) 0.26; ν_max_ (neat) 1026, 1074, 1347, 1441, 1481, 1549, 2923 cm^-1^; ^1^H-NMR δ_H_ (300 MHz, CDCl_3_) 6.86–6.90 (m, 2H), 6.97–7.01 (m, 3H), 7.11–7.15 (m, 2H), 7.19–7.22 (m, 3H), 7.25–7.30 (m, 3H), 7.35–7.39 (m, 2H), 7.45 (dt, *J* 1.5 and 7.4 Hz, 1H), 7.58 (td, *J* 0.6 and 8.4 Hz, 1H), 7.73 (dt, *J* 1.5 and 7.4 Hz, 1H), 8.26 (dd, *J* 0.6 and 8.4 Hz, 1H); ^13^C-NMR δ_C_ (75 MHz, CDCl_3_) 126.3, 126.5, 126.6, 126.7, 127.2, 127.3, 127.5, 127.7, 127.8, 129.3, 129.7, 129.9, 130.3, 131.3, 132.9, 136.9, 138.3, 141.1, 147.3, 147.6, 159.0; MS *m/z* (100, MH^+^) 358; HRMS (ES): MH^+^, found 358.1585. C_27_H_20_N^+^ requires 358.1596.

2*-(4-Fluorophenyl)-3,4-diphenylquinoline* (**3b**). A mixture of **1b** (0.50 g, 1.30 mmol), phenylboronic acid (0.40 g, 3.26 mmol), PdCl_2_(PPh_3_)_2_ (0.05 g, 0.07 mmol), PCy_3_ (0.04 g, 0.13 mmol), and K_2_CO_3_ (0.36 g, 2.61 mmol) in dioxane/water (20 mL) afforded **(3b**) as a solid (0.27 g, 55%), mp 181–183 °C (ethanol); *R_f_* (10% ethyl acetate/hexane) 0.38; ν_max_ (neat) 836, 1158, 1232, 1345, 1479, 1509, 1601, 3052 cm^−1^; ^1^H-NMR δ_H_ (300 MHz, CDCl_3_) 6.86–6.92 (m, 4H), 7.00–7.05 (m, 3H), 7.11–7.15 (m, 2H), 7.24–7.30 (m, 3H), 7.36 (dd, *J* 5.4 and 9.0 Hz, 2H), 7.45 (dt, *J* 1.2 and 7.8 Hz, 1H), 7.58 (dd, *J* 1.5 and 8.4 Hz, 1H), 7.73 (dt, *J* 1.2 and 7.8 Hz, 1H), 6.23 (d, *J* 8.4 Hz, 1H); ^13^C-NMR δ_C_ (75 MHz, CDCl_3_) 114.6 (d, ^2^*J*_CF_ 21.9 Hz), 126.4, 126.6, 126.7 (2xC), 127.3, 127.5, 127.8, 129.5, 129.6, 130.2, 131.3, 131.8 (d, ^3^*J*_CF_ 8.3 Hz), 132.8, 136.8, 137.2 (d, ^4^*J*_CF_ 3.4 Hz), 138.2, 147.3, 147.8, 157.8, 162.4 (d, ^1^*J*_CF_ 245.9 Hz); MS *m/z* (100, MH^+^) 376; HRMS (ES): MH^+^, found 376.1491. C_27_H_19_FN^+^ requires 376.1502.

2*-(4-Chlorophenyl)-3,4-diphenylquinoline* (**3c**). A mixture of **1c** (0.30 g, 0.75 mmol), phenylboronic acid (0.23 g, 1.88 mmol), PdCl_2_(PPh_3_)_2_ (0.03 g, 0.04 mmol), PCy_3_ (0.02 g, 0.08 mmol), and K_2_CO_3_ (0.21 g, 1.50 mmol) in dioxane/water (11 mL) afforded (**3c**) as a solid (0.18 g, 61%), mp 148–151 °C (ethanol); *R_f_* (10% ethyl acetate–hexane) 0.46; ν_max_ (neat) 833, 1014, 1093, 1347, 1482, 1546, 2926 cm^−1^; ^1^H-NMR δ_H_ (300 MHz, CDCl_3_) 6.85–6.89 (m, 2H), 7.00–7.04 (m, 3H), 7.09–7.13 (m, 2H), 7.32 (d, *J* 8.4 Hz, 2H), 7.24–7.28 (m, 3H), 7.32 (d, *J* 8.4 Hz, 2H), 7.45 (t, *J* 8.4 Hz, 1H), 7.57 (d, *J* 7.5 Hz, 1H), 7.73 (t, *J* 7.5 Hz, 1H), 8.22 (d, *J* 8.4 Hz, 1H); ^13^C-NMR δ_C_ (75 MHz, CDCl_3_) 126.5, 126.6, 126.7, 126.8, 127.4, 127.6, 127.8, 127.9, 129.5, 129.7, 130.2, 131.2, 131.3, 132.7, 133.8, 136.7, 138.0, 139.6, 147.3, 147.9, 157.6; MS *m/z* (100, MH^+^) 392; HRMS (ES): MH^+^, found 392.1200. C_27_H_19_N^35^Cl^+^ requires 392.1206.

*2-(4-Methoxyphenyl)-3,4-diphenylquinoline* (**3d**). A mixture of **1d** (0.30 g, 0.77 mmol), phenylboronic acid (0.24 g, 1.93 mmol), PdCl_2_(PPh_3_)_2_ (0.03 g, 0.04 mmol), PCy_3_ (0.02 g, 0.08 mmol), and K_2_CO_3_ (0.21 g, 1.55 mmol) in dioxane/water (20 mL) afforded (**3d**) as a solid (0.17 g, 58%), mp 177–179 °C (ethanol); *R_f_* (30% ethyl acetate/hexane) 0.79; ν_max_ (neat) 831, 1026, 1248, 1514, 1607 cm^-1^; ^1^H-NMR δ_H_ (300 MHz, CDCl_3_) 3.76 (s, 3H), 6.73 (d, *J* 9.3 Hz, 2H), 6.87–6.92 (m, 2H), 7.00–7.03 (m, 3H), 7.10–7.13 (m, 2H), 7.24–7.28 (m, 3H), 7.35 (d, *J* 8.4 Hz, 2H), 7.42 (t, *J* 7.5 Hz, 1H), 7.55 (d, *J* 8.4 Hz, 1H), 7.71 (t, *J* 8.4 Hz, 1H), 8.23 (d, *J* 8.4 Hz, 1H); ^13^C-NMR δ_C_ (75 MHz, CDCl_3_) 55.2, 113.1, 126.2, 126.3, 126.5, 126.6, 127.2, 127.4, 127.7, 129.2, 129.6, 130.3, 131.3 (2xC), 132.8, 133.6, 137.0, 138.6, 147.3, 147.6, 158.4, 159.2; MS *m/z* (100, MH^+^) 388; HRMS (ES): MH^+^, found 388.1711. C_28_H_22_NO^+^ requires 388.1701.

*3,4-Bis(4-fluorophenyl)-2-phenylquinoline* (**3e**). A mixture of **1a** (0.50 g, 1.37 mmol), 4-fluorophenylboronic acid (0.48 g, 3.42 mmol), PdCl_2_(PPh_3_)_2_ (0.05 g, 0.07 mmol), PCy_3_ (0.04 g, 0.14 mmol), and K_2_CO_3_ (0.38 g, 2.74 mmol) in dioxane/water (20 mL) afforded (**3e**) as a solid (0.39 g, 72%), mp 183–185 °C (ethanol); *R_f_* (10% ethyl acetate/hexane) 0.27; ν_max_ (neat) 839, 1224, 1487, 1511, 1605, 3059 cm^-1^; ^1^H-NMR δ_H_ (300 MHz, CDCl_3_) 6.74 (t, *J* 8.7 Hz, 2H), 6.80–6.86 (m, 2H), 7.01 (t, *J* 8.7 Hz, 2H), 7.07–7.12 (m, 2H), 7.22–7.26 (m, 3H), 7.33–7.37 (m, 2H), 7.48 (dt, *J* 1.2 and 7.5 Hz, 1H), 7.56 (td, *J* 1.2 and 8.4 Hz, 1H), 7.75 (dt, *J* 1.5 and 7.8 Hz, 1H), 8.26 (d, *J* 8.4 Hz, 1H); ^13^C-NMR δ_C_ (75 MHz, CDCl_3_) 114.6 (d, ^2^*J*_CF_ 21.4 Hz), 115.1 (d, ^2^*J*_CF_ 21.4 Hz), 126.3, 126.6, 126.8, 127.7, 127.8, 129.6, 129.8 (2xC), 131.9 (d, ^3^*J*_CF_ 8.3 Hz), 132.1, 132.6 (d, ^4^*J*_CF_ 3.5 Hz), 132.8 (d, ^3^*J*_CF_ 8.3 Hz,), 134.1 (d, ^4^*J*_CF_ 3.4 Hz), 140.9, 146.8, 147.4, 159.0, 161.3 (d, ^1^*J*_CF_ 245.3 Hz), 162.0 (d, ^1^*J*_CF_ 245.9 Hz); MS *m/z* (100, MH^+^) 394; HRMS (ES): MH^+^, found 394.1389. C_27_H_18_F_2_N^+^ requires 394.1407.

*2,3,4-Tris(4-fluorophenyl)quinoline* (**3f**)*.* A mixture of **1b** (0.20 g, 0.52 mmol), 4-fluorophenylboronic acid (0.18 g, 1.30 mmol), PdCl_2_(PPh_3_)_2_ (0.02 g, 0.03 mmol), PCy_3_ (0.01 g, 0.05 mmol), and K_2_CO_3_ (0.14 g, 1.04 mmol) in dioxane/water (12 mL) afforded (**3f**) as a solid (0.153 g, 75%), mp 158–163 °C (ethanol); *R_f_* (10% ethyl acetate/hexane) 0.27; ν_max_ (neat) 833, 1157, 1219, 1509, 1601 cm^−1^; ^1^H-NMR δ_H_ (300 MHz, CDCl_3_) 6.75 (t, *J* 8.7 Hz, 2H), 6.77–6.85 (m, 2H), 6.92 (t, *J* 8.7 Hz, 2H), 7.00 (t, *J* 8.7 Hz, 2H), 7.05–7.11 (m, 2H), 7.31–7.36 (m, 2H), 7.48 (dt, *J* 1.2 and 7.5 Hz, 1H), 7.53 (t, *J* 1.2 and 7.5 Hz, 1H), 7.75 (dt, *J* 1.5 and 7.8 Hz, 1H), 8.23 (d, *J* 8.7 Hz, 1H); ^13^C-NMR δ_C_ (75 MHz, CDCl_3_) 114.8 (d, ^2^*J*_CF_ 21.4 Hz, 2xC), 115.2 (d, ^2^*J*_CF_ 21.4 Hz), 126.3, 126.6, 126.9, 129.7, 129.8 (2xC), 131.7 (d, ^3^*J*_CF_ 8.3 Hz), 131.8, 131.9 (d, ^3^*J*_CF_ 8.3 Hz), 132.0, 132.5 (d, ^4^*J*_CF_ 3.5 Hz), 132.8 (d, ^3^*J*_CF_ 8.4 Hz), 134.0 (d, ^4^*J*_CF_ 3.5 Hz), 136.9 (d, ^4^*J*_CF_ 3.4 Hz), 157.8, 161.4 (d, ^1^*J*_CF_ 245.6 Hz), 162.0 (d, ^1^*J*_CF_ 246.2 Hz), 162.5 (d, ^1^*J*_CF_ 246.4 Hz); MS *m/z* (100, MH^+^) 412; HRMS (ES): MH^+^, found 412.1314. C_27_H_17_F_3_N^+^ requires 412.1313.

*2-(4-Chlorophenyl)-3,4-bis(4-fluorophenyl)quinoline* (**3g**). A mixture of **1c** (0.30 g, 0.75 mmol), 4-fluorophenylboronic acid (0.26 g, 1.88 mmol), PdCl_2_(PPh_3_)_2_ (0.03 g, 0.04 mmol), PCy_3_ (0.02 g, 0.08 mmol), and K_2_CO_3_ (0.21 g, 1.50 mmol) in dioxane/water (12 mL) afforded (**3g**) as a solid (0.20 g, 62%), mp 183–185 °C (ethanol); *R_f_* (10% ethyl acetate/hexane) 0.29; ν_max_ (neat) 832, 1093, 1157, 1223, 1509, 1604 cm^−1^; ^1^H-NMR δ_H_ (300 MHz, CDCl_3_) 6.72–6.85 (m, 4H), 6.97–7.10 (m, 4H), 7.21 (d, *J* 9.0 Hz, 2H), 7.29 (d, *J* 9.0 Hz, 2H), 7.45–7.56 (m, 2H), 7.75 (dt, *J* 1.8 and 7.5 Hz, 1H), 8.23 (dd, *J* 0.9 and 8.4 Hz, 1H); ^13^C-NMR δ_C_ (75 MHz, CDCl_3_) 114.9 (d, ^2^*J*_CF_ 21.3 Hz), 115.2 (d, ^2^*J*_CF_ 21.7 Hz), 126.3, 126.6, 127.1, 128.1, 129.7, 129.8, 131.2 (2xC), 131.8 (d, ^3^*J*_CF_ 8.0 Hz), 132.4 (d, ^4^*J*_CF_ 3.4 Hz), 132.8 (d, ^3^*J*_CF_ 8.1 Hz), 133.9 (d, ^4^*J*_CF_ 3.5 Hz), 134.0, 139.3, 147.1, 147.4, 157.9, 161.4 (d, ^1^*J*_CF_ 245.9 Hz), 162.0 (d, ^1^*J*_CF_ 245.9 Hz); MS *m/z* (100, MH^+^) 428; HRMS (ES): MH^+^, found 428.0999. C_27_H_17_F_2_N^35^Cl^+^ requires 428.1018.

*3,4-Bis(4-fluorophenyl)-2-(4-methoxyphenyl)quinoline* (**3h**). A mixture of **1d** (0.30 g, 0.76 mmol), 4-fluorophenylboronic acid (0.27 g, 1.89 mmol), PdCl_2_(PPh_3_)_2_ (0.03 g, 0.04 mmol), PCy_3_ (0.02 g, 0.08 mmol), and K_2_CO_3_ (0.21 g, 1.52 mmol) in dioxane/water (12 mL) afforded (**3h**) as a solid (0.20 g, 62%), mp 169–182 °C (ethanol); *R_f_* (30% ethyl acetate/hexane) 0.79; ν_max_ (neat) 829, 1222, 1251, 1510, 1604 cm^−1^; ^1^H-NMR δ_H_ (300 MHz, CDCl_3_) 3.76 (s, 3H), 6.76 (dd, *J* 1.5 and 8.7 Hz, 4H), 6.84 (dd, *J* 5.4 and 8.7 Hz, 2H), 6.99 (t, *J* 8.7 Hz, 2H), 7.08 (dd, *J* 5.4 and 8.7 Hz, 2H), 7.31 (d, *J* 9.0 Hz, 2H), 7.44 (dt, *J* 1.5 and 7.8 Hz, 1H), (td, *J* 0.9 and 8.7 Hz, 1H), 7.72 (dt, *J* 1.8 and 7.5 Hz, 1H), 8.23 (dd, *J* 0.6 and 7.8 Hz, 1H); ^13^C-NMR δ_C_ (75 MHz, CDCl_3_) 55.2, 113.3, 114.7 (d, ^2^*J*_CF_ 21.4 Hz), 115.1 (d, ^2^*J*_CF_ 21.4 Hz), 126.3, 126.4, 126.6, 129.5, 129.7, 131.3, 131.9 (d, ^3^*J*_CF_ 8.3 Hz), 132.0 (d, ^4^*J*_CF_ 3.4 Hz), 132.8 (d, ^3^*J*_CF_ 8.0 Hz), 133.3, 134.4 (d, ^3^*J*_CF_ 3.7 Hz), 146.7, 147.4, 158.4, 159.2 (2xC), 161.3 (d, ^1^*J*_CF_ 245.6 Hz), 161.9 (d, ^1^*J*_CF_ 246.2 Hz); MS *m/z* (100, MH^+^) 424; HRMS (ES): MH^+^, found 424.1499. C_28_H_20_F_2_NO^+^ requires 424.1513.

### 3.3. Synthesis of 2-aryl-4-chloro-3-(4-fluorophenyl)quinolines 2e-h. typical procedure

A mixture of 2-aryl-4-chloro-3-iodoquinoline **1** (1 equiv.), arylboronic acid (1.2 equiv.) and Pd(PPh_3_)_4_ (5% of **1**) in DMF (5 mL/mmol of **1**) in a two-necked flask equipped with a stirrer bar, rubber septum and a condenser was flushed with nitrogen gas. After 10 minutes 2M K_2_CO_3_ (2 mL/mmol of **1**) was added and the mixture was flushed for additional 10 minutes with nitrogen gas. A balloon filled with nitrogen gas was connected to the top of the condenser and the mixture was heated with stirring at 80–90 °C for 48 hours. The mixture was allowed to cool to room temperature and then quenched with ice-cold water. The product was extracted with chloroform and the combined organic extracts were washed with brine, dried over anhydrous MgSO_4_, filtered and then evaporated under reduced pressure. The residue was purified by column chromatography to afford the 2-aryl-4-chloro-3-(4-fluorophenyl)quinoline **2**. The following products were prepared:

*4-Chloro-3-(4-fluorophenyl)-2-phenylquinoline* (**2e**). A mixture of **1a** (0.55 g, 1.50 mmol), 4-fluorophenylboronic acid (0.25 g, 1.81 mmol), Pd(PPh_3_)_4_ (0.09 g, 0.08 mmol), and 2M K_2_CO_3_ (3 mL) in DMF (8 mL) afforded (**2e**) as a solid (0.30 g, 60%), mp 147–149 °C (ethanol); *R_f_* (10% ethyl acetate/hexane) 0.42; ν_max_ (neat) 839, 1157, 12111, 1337, 1337, 1475, 1507, 1565; ^1^H-NMR δ_H_ (300 MHz, CDCl_3_) 7.01 (t, *J* 9.0 Hz, 2H), 7.13–7.18 (m, 2H), 7.20–7.26 (m, 3H), 7.28–7.33 (m, 2H), 7.67 (dt, *J* 1.5 and 7.8 Hz, 1H), 7.80 (dt, *J* 1.5 and 7.4 Hz, 1H), 8.20 (d, *J* 2.4 and 7.5 Hz, 1H), 8.31 (dt, *J* 0.3 and 8.7 Hz, 1H); ^13^C-NMR δ_C_ (75 MHz, CDCl_3_) 115.2 (d, ^2^*J*_CF_ 21.7Hz), 124.7, 125.4, 127.8, 127.9, 128.1, 129.7, 130.5, 132.0, 132.5 (d, ^3^*J*_CF_ 8.3 Hz), 132.9 (d, ^4^*J*_CF_ 3.5 Hz), 140.1, 142.1, 147.7, 159.2, 162.2 (d, ^1^*J*_CF_ 246.5 Hz); MS *m/z* (100, MH^+^) 334; HRMS (ES): MH^+^, found 334.0817. C_21_H_14_FN^35^Cl^+^ requires 334.0799.

*4-Chloro-2,3-bis(4-fluorophenyl)quinoline* (**2f**). A mixture of **1b** (0.50 g, 1.30 mmol), 4-fluorophenylboronic acid (0.22 g, 1.56 mmol), Pd(PPh_3_)_4_ (0.08 g, 0.07 mmol), and 2M K_2_CO_3_ (2.6 mL) in DMF (7 mL) afforded (**2f**) as a solid (0.25 g, 55%), mp 183–185 °C (ethanol); *R_f_* (10% ethyl acetate/hexane) 0.42; ν_max_ (neat) 831, 1158, 1219, 1337, 1474, 1509, 1597 cm^−1^; ^1^H-NMR δ_H_ (300 MHz, CDCl_3_) 6.92 (t, *J* 8.7 Hz, 2H), 7.04 (t, *J* 8.7 Hz, 2H), 7.16 (dd, *J* 5.4 and 8.8 Hz, 2H), 7.30 (dd, *J* 5.4 and 8.8 Hz, 2H), 7.68 (dt, *J* 1.2 and 7.8 Hz, 1H*),* 7.81 (dt, *J* 1.2 and 7.8 Hz, 1H*)*, 8.19 (dddd, *J* 0.6, 1.2 and 8.4 Hz, 1H), (dddd, *J* 0.6, 1.6 and 8.4 Hz, 1H); ^13^C-NMR δ_C_ (75 MHz, CDCl_3_) 114.9 (d, ^2^*J*_CF_ 21.4 Hz), 115.3 (d, ^2^*J*_CF_ 21.6 Hz), 124.7, 125.4, 127.9, 129.8, 130.6, 131.6 (d, ^3^*J*_CF_ 8.3 Hz), 131.8, 132.4 (d, ^3^*J*_CF_ 8.3 Hz), 132.8 (d, ^4^*J*_CF_ 3.4 Hz), 136.1 (d, ^4^*J*_CF_ 3.4 Hz), 142.3, 147.6, 158.0, 162.2 (d, ^1^*J*_CF_ 246.8 Hz), 162.6 (d, ^1^*J*_CF_ 247.0 Hz); MS *m/z* (100, MH^+^) 352; HRMS (ES): MH^+^, found 352.0709. C_21_H_13_F_2_N^35^Cl^+^ requires 352.0705.

*4-Chloro-2-(4-chlorophenyl)-3-(4-fluorophenyl)quinoline* (**2g**). A mixture of **1c** (0.50 g, 1.25 mmol), 4-fluorophenylboronic acid (0.21 g, 1.50 mmol), Pd(PPh_3_)_4_ (0.07 g, 0.07 mmol), and 2M K_2_CO_3_ (2.5 mL) in DMF (6.5 mL) afforded (**2g**) as a solid (0.28 g, 61%), mp 168–171 °C (ethanol); *R_f_* (10% ethyl acetate–hexane) 0.51; ν_max_ (neat) 827, 1092, 1341, 1474, 1509 cm^-1^; ^1^H-NMR δ_H_ (300 MHz, CDCl_3_) 7.04 (t, *J* 8.4 Hz, 2H), 7.13–7.28 (m, 6H), 7.69 (dt, *J* 1.2 and 7.8 Hz, 1H), 7.81 (dt, *J* 1.2 and 7.8 Hz, 1H), 8.18 (dd, *J* 0.6 and 7.8 Hz, 1H), 8.31 (td, *J* 0.9 and 8.4 Hz, 1H); ^13^C-NMR δ_C_ (75 MHz, CDCl_3_) 115.4 (d, ^2^*J*_CF_ 21.6 Hz), 124.7, 125.5, 128.0, 128.1, 129.9, 130.6, 131.1, 131.8, 132.4, (d, ^3^*J*_CF_ 8.3 Hz), 132.6 (d, ^4^*J*_CF_ 3.4 Hz), 134.4, 138.6, 142.3, 147.7, 157.8, 162.3 (d, ^1^*J*_CF_ 246.45 Hz); MS *m/z* (100, MH^+^) 368; HRMS (ES): MH^+^, found 368.0395. C_21_H_13_FN^35^Cl_2_^+^ requires 368.0409.

*4-Chloro-3-(4-fluorophenyl)-2-(4-methoxyphenyl)quinoline* (**2h**). A mixture of **1d** (0.50 g, 1.26 mmol), 4-fluorophenylboronic acid (0.21 g, 1.52 mmol), Pd(PPh_3_)_4_ (0.07 g, 0.06 mmol), and 2M K_2_CO_3_ (2.5 mL) in DMF (7 mL) afforded (**2h**) as a solid (0.36 g, 79%), mp 155–157 °C (ethanol); *R_f_* (10% ethyl acetate/hexane) 0.23; ν_max_ (neat) 828, 1032, 1175, 1245, 1337, 1513, 1607, 2835 cm^−1^; ^1^H-NMR δ_H_ (300 MHz, CDCl_3_) 3.78 (s, 3H), 6.76 (dd, *J* 2.1 and 8.7 Hz, 2H), 7.04 (t, *J* 8.4 Hz, 2H), 7.14–7.21 (m, 2H), 7.28 (d, *J* 2.1 and 8.7 Hz, 2H), 7.65 (dt, *J* 1.2 and 7.8 Hz, 1H), 7.78 (dt, *J* 1.2 and 7.5 Hz, 1H), 8.19 (d, *J* 8.1 Hz, 1H), 8.24 (dd, *J* 0.9 and 8.4 Hz, 1H); ^13^C-NMR δ_C_ (75 MHz, CDCl_3_) 55.2, 113.3, 115.2 (d, ^2^*J*_CF_ 21.4 Hz), 124.6, 125.2, 127.5, 129.6, 131.2, 131.8, 132.5 (d, ^3^*J*_CF_ 8.3 Hz), 133.2 (d, ^4^*J*_CF_ 3.4 Hz), 142.0, 147.7, 158.7, 159.5, 159.5, 162.1 (d, ^1^*J*_CF_ 246.2 Hz); MS *m/z* (100, MH^+^) 364; HRMS (ES): MH^+^, found 364.0905. C_22_H_16_FNO^35^Cl^+^ requires 364.0904.

### 3.4. Reaction of 2e-h with aniline. typical procedure

A mixture of **2** (1 equiv.) and aniline (2.5 equiv.) was heated under reflux for 18 hours. The cooled mixture was quenched with ice-cold water and then extracted with chloroform. The combined organic phase was dried over MgSO_4_, filtered and then evaporated under reduced pressure. The residue was purified by column chromatography to afford (**4**).

*3-(4-Fluorophenyl)-2-phenyl-4-(phenylamino)quinoline* (**4a**). A mixture of **2e** (0.08 g, 0.24 mmol) and aniline (0.06 g, 0.60 mmmol) afforded (**4a**) as a solid (0.05 g, 53%), mp 189–192 °C (ethanol); *R_f_* (30% ethyl acetate/hexane) 0.64; ν_max_ (neat) 744, 833, 1213, 1234, 1372, 1399, 1490, 1573, 3393 cm^−1^; ^1^H-NMR δ_H_ (300 MHz, CDCl_3_) 5.80 (br s, 1H), 6.76 (d, *J* 7.8 Hz, 2H), 6.96 (t, *J* 8.7 Hz, 3H), 7.06–7.11 (m, 2H), 7.18–7.25 (m, 5H), 7.29–7.33 (m, 2H), 7.34 (dt, *J* 1.5 and 7.7 Hz, 1H), 7.67 (dt, *J* 1.5 and 7.7 Hz, 1H), 7.77 (dd, *J* 0.6 and 8.4 Hz, 1H), 8.17 (dd, *J* 0.6 and 8.4 Hz, 1H); ^13^C-NMR δ_C_ (75 MHz, CDCl_3_) 116.0 (d, ^2^*J*_CF_ 21.3 Hz), 118.3, 121.8, 121.9, 124.7, 125.2, 125.6, 127.7, 127.8, 129.3, 129.6, 129.7, 130.1, 131.8 (d, ^4^*J*_CF_ 3.8 Hz), 132.4 (d, ^3^*J*_CF_ 8.0 Hz), 140.9, 145.0, 145.1, 148.6, 159.5, 162.2 (d, ^1^*J*_CF_ 246.5 Hz); MS *m/z* (100, MH^+^) 391; HRMS (ES): MH^+^, found 391.1611. C_27_H_20_FN_2_^+^ requires 391.1617.

*2,3-Bis(4-fluorophenyl)-4-(phenylamino)quinoline* (**4b**). A mixture of **2f** (0.05 g, 0.14 mmol) and aniline (0.03g, 0.35 mmol) afforded (**4b**) as a solid (0.03 g, 52%), mp 178–181 °C (ethanol); *R_f_* (30% ethyl acetate/hexane) 0.70; ν_max_ (neat) 748, 758, 834, 946, 1214, 1232, 1491, 1509, 1575, 1599, 3391 cm^−1^; ^1^H-NMR δ_H_ (300 MHz, CDCl_3_) 5.80 (s, 1H), 6.77 (d, *J* 7.8 Hz, 2H), 6.91 (t, *J* 8.7 Hz, 2H), 6.94–7.02 (m, 3H), 7.06–7.12 (m, 2H), 7.20 (t, *J* 7.8 Hz, 2H), 7.27–7.33 (m, 2H), 7.34 (dt, *J* 1.2 and 7.5 Hz, 1H), 7.67 (dt, *J* 1.5 and 7.4 Hz, 1H), 7.76 (dd, *J* 0.6 and 8.6 Hz, 1H), 8.14 (dd, *J* 0.6 and 8.7 Hz, 1H); ^13^C-NMR δ_C_ (75 MHz, CDCl_3_) 114.8 (d, ^2^*J*_CF_ 21.4 Hz), 116.2 (d, ^2^*J*_CF_ 21.4 Hz), 118.3, 121.7, 122.0, 124.4, 125.2, 125.6, 129.3, 129.7, 130.0, 131.5 (d, ^3^*J*_CF_ 8.0 Hz), 131.6 (d, ^4^*J*_CF_ 3.7 Hz), 132.3 (d, ^3^*J*_CF_ 8.0 Hz), 136.8 (d, ^4^*J*_CF_ 3.2 Hz), 145.0, 145.2, 148.5, 158.3, 162.3 (d, ^1^*J*_CF_ 247.0 Hz), 162.4 (d, ^1^*J*_CF_ 246.2 Hz); MS *m/z* (100, MH^+^) 409; HRMS (ES): MH^+^, found 409.1523. C_27_H_19_F_2_N_2_^+^ requires 409.1516.

*2-(4-Chlorophenyl)-3-(4-fluorophenyl)-4-(phenylamino)quinoline* (**4c**). A mixture of **2g** (0.10 g, 0.27 mmol) and aniline (0.06 g, 0.66 mmol) afforded (**4c**) as solid (0.08 g, 69%), mp 200–203 °C (ethanol); *R_f_* (3:7, ethyl acetate/hexane) 0.74; ν_max_ (neat) 747, 762, 831, 1091, 1218, 1400, 1498, 1569, 3391 cm^−1^; ^1^H-NMR δ_H_ (300 MHz, CDCl_3_) 5.85 (br s, 1H), 6.79 (d, *J* 9.0 Hz, 2H), 7.01 (t, *J* 8.4 Hz, 3H), 7.07–7.13 (m, 2H), 7.18–7.29 (m, 6H), 7.36 (dt, *J* 1.5 and 7.7 Hz, 1H), 7.69 (dt, *J* 1.5 and 7.7 Hz, 1H), 7.76 (dd, *J* 0.6 and 8.4 Hz, 1H), 8.16 (d, *J* 8.4 Hz, 1H); ^13^C-NMR δ_C_ (75 MHz, CDCl_3_) 116.3 (d, ^2^*J*_CF_ 21.3 Hz), 118.5, 121.6, 122.1, 124.3, 125.2, 125.7, 128.0, 129.3, 129.9, 131.0, 131.4 (d, ^4^*J*_CF_ 3.8 Hz), 132.4 (d, ^3^*J*_CF_ 8.0 Hz), 133.9, 139.1, 144.8 (2xC), 145.4, 148.4, 158.1, 162.2 (d, ^1^*J*_CF_ 247.2 Hz); *m/z* (100, MH^+^) 425; HRMS (ES): MH^+^, found 425. 1313. C_27_H_19_FN_2_^35^Cl^+^ requires 425. 1315.

*3-(4-Fluorophenyl)-2-(4-methoxyphenyl)-4-(phenylamino)quinoline* (**4d**). A mixture of **2h** (0.10 g, 0.28 mmol) and aniline (0,07 g, 0.70 mmol) afforded (**4d**) as a solid (0.07 g, 61%), mp 180–182 °C (ethanol); *R_f_* (30% ethyl acetate/hexane) 0.57; ν_max_ (neat) 767, 834, 1026, 1214, 1243, 1399, 1492, 1508, 1573, 3388 cm^-1^; ^1^H-NMR δ_H_ (300 MHz, CDCl_3_) 3.77 (s, 3H), 5.78 (s, 1H), 6.73–6.77 (m, 4H), 6.93–7.01 (m, 3H), 7.07–7.12 (m, 2H), 7.19 (t, *J* 7.8 Hz, 2H), 7.26 (d, *J* 8.7 Hz, 2H), 7.32 (dt, *J* 1.2 and 7.5 Hz, 1H), 7.65 (dt, *J* 1.5 and 7.4 Hz, 1H), 7.75 (dd, *J* 0.6 and 8.6 Hz, 1H), 8.14 (dd, *J* 0.6 and 8.7 Hz, 1H); ^13^C-NMR δ_C_ (75 MHz, CDCl_3_) 55.2, 113.2, 116.1 (d, ^2^*J*_CF_ 21.4 Hz), 118.1, 121.6, 121.7, 124.7, 125.2, 125.4, 129.2, 129.5, 130.0, 131.1, 132.0 (d, ^4^*J*_CF_ 3.4 Hz), 132.3 (d, ^3^*J*_CF_ 8.0 Hz), 133.3, 144.9, 145.2, 148.5, 159.0, 159.2, 162.2 (d, ^1^*J*_CF_ 246.2 Hz); MS *m/z* (100, MH^+^) 421; HRMS (ES): MH^+^, found 421.1722. C_28_H_22_N_2_FO^+^ requires 421.1716.

### 3.5. Hydrolysis of 4 with acetic acid: typical procedure

A suspension of **2** (1 equiv.) in acetic acid-water (5:1, v/v) was refluxed for 6 hours. The mixture was quenched with ice-cold water and the precipitate was filtered and recrystallized to afford **5**.

*3-(4-Fluorophenyl)-2-phenylquinolin-4(1H)-one* (**5a**). A suspension of **2e** (0.06 g, 0.18 mmol) in 5:1 acetic acid-water (10 mL) afforded (**5a**) as a solid (0.04 g, 70%), mp 340–342 °C (ethanol); ν_max_ (neat) 1213, 1352, 1495, 1251, 1552, 1624, 3095 cm^−1^; ^1^H-NMR δ_H_ (300 MHz, DMSO-*d*_6_) 6.78 (t, *J* 9.0 Hz, 2H), 7.02 (dd, *J* 6.0 and 8.4 Hz, 2H), 7.24 (s, 5H), 7.26 (d, *J* 7.8 Hz, 1H), 7.51 (t, *J* 7.5 Hz, 1H), 7.62 (d, *J* 7.8 Hz, 1H), 8.21 (d, *J* 7.8 Hz, 1H), 11.54 (br s, 1H); ^13^C-NMR δ_C_ (75 MHz, DMSO*d*_6_) 114.2 (d, ^2^*J*_CF_ 21.1 Hz), 118.5, 119.8, 123.2, 125.0, 125.7, 128.1, 129.0, 129.6, 131.5 (d, ^4^*J*_CF_ 3.4 Hz), 131.6, 133.3 (d, ^3^*J*_CF_ 8.1 Hz), 135.4, 139.9, 148.6, 161.0 (d, ^1^*J*_CF_ 242.8 Hz), 176.4; MS *m/z* (100, MH^+^) 316; HRMS (ES): MH^+^, found 316.1138. C_21_H_15_FNO^+^ requires 316.1125.

*2,3-Bis(4-fluorophenyl)quinolin-4(1H)-one* (**5b**). A suspension of **2f** (0.06 g, 0.171 mmol) in acetic acid-water (10 mL) afforded **5b** as a solid (0.04 g, 70%), mp 347–349 °C (ethanol); ν_max_ (neat) 829, 1159, 1221, 1351, 1351, 1500, 1521, 1604, 1625, 3065 cm^−1^; ^1^H-NMR δ_H_ (300 MHz, DMSO-*d*_6_) 7.01 (t, *J* 9.0 Hz, 2H), 7.041–7.11 (m, 2H), 7.21 (t, *J* 9.0 Hz, 2H), 7.33–7.41 (m, 3H), 7.68 (d, *J* 3.0 Hz, 2H), 8.15 (d, *J* 9.0 Hz, 1H,), 11.85 (br s, 1H); ^13^C-NMR δ_C_ (75 MHz, CDCl_3_) 114.7 (d, ^2^*J*_CF_ 20.8 Hz), 115.7 (d, ^2^*J*_CF_ 21.6 Hz), 118.9, 120.0, 123.8, 125.1, 125.8, 131.9 (d, ^4^*J*_CF_ 3.2 Hz), 132.3 (d, ^4^*J*_CF_ 3.1 Hz), 132.3, 132.5 (d, ^3^*J*_CF_ 8.6 Hz), 134.0 (d, ^3^*J*_CF_ 8.0 Hz), 140.1, 148.2, 161.1 (d, ^1^*J*_CF_ 241.4 Hz), 162.7 (d, ^1^*J*_CF_ 245.0 Hz), 175.8; MS *m/z* (100, MH^+^) 334; HRMS (ES): MH^+^, found 334.1046. C_21_H_14_F_2_NO^+^ requires 334.1043.

*2-(4-Chlorophenyl)-3-(4-fluorophenyl)quinolin-4(1H)-one* (**5c**). A suspension of **2g** (0.06 g, 0.172 mmol) in acetic acid-water (10 mL) afforded **5c** as a solid (0.03 g, 55%), mp 309–312 °C (ethanol); ν_max_ (neat) 822, 1091, 1210, 1350, 1491, 1519, 1551, 1600, 1624, 3089 cm^−1^; ^1^H-NMR δ_H_ (300 MHz, DMSO-*d*_6_) 6.81 (t, *J* 9.0 Hz, 2H), 7.02 (dd, *J* 6.0 and 8.4 Hz, 2H), 7.18 (d, *J* 9.0 Hz, 2H), 7.23 (d, *J* 9.0 Hz, 2H), 7.51 (t, *J* 7.5 Hz, 1H), 7.56 (t, *J* 7.5 Hz, 2H), 8.23 (d, *J* 7.8 Hz, 1H), 11.42 (br s, 1H); ^13^C-NMR δ_C_ (75 MHz, CDCl_3_) 114.5 (d, *^2^J*_CF_ 21.1 Hz), 118.3, 119.9, 123.3, 125.0, 125.8, 128.3, 131.1, 131.7, 131.8 (d, ^4^*J*_CF_ 3.2 Hz), 133.3 (d, ^3^*J*_CF_ 8.0 Hz), 133.9, 134.8, 139.8, 147.2, 161.2 (d, ^1^*J*_CF_ 243.1 Hz), 176.5; MS *m/z* (100, MH^+^) 350; HRMS (ES): MH^+^, found 350.0748. C_21_H_14_F_2_NO^35^Cl^+^ requires 350.0748.

*3-(4-Fluorophenyl)-2-(4-methoxyphenyl)quinolin-4(1H)-one* (**5d**). A suspension of **2h** (0.10 g, mmol) in acetic acid (5 mL) afforded (**5d**) as a solid (0.05 g, 65%), mp 375–377 °C (ethanol); ^1^H-NMR δ_H_ (300 MHz, DMSO-*d*_6_) 3.75 (s, 3H), 6.90 (d, *J* 9.0 Hz, 2H), 6.98–7.11 (m, 4H), 7.23 (d, *J* 9.0 Hz, 2H), 7.34 (t, *J* 7.5 Hz, 1H), 7.67 (1H, *J* 7.5 Hz, 1H ), 7.68 (d, *J* 7.5 Hz, 1H), 8.13 (d, *J* 7.8 Hz, 1H), 11.78 (br s, 1H); ^13^C-NMR δ_C_ (75 MHz, DMSO-*d*_6_) 55.6, 113.9, 114.4 (d, ^2^*J*_CF_ 21.1 Hz), 118.8, 119.8, 123.5, 125.0, 125.7, 127.6, 131.4, 131.6 (d, ^4^*J*_CF_ 3.4 Hz), 132.0, 133.9 (d, ^3^*J*_CF_ 8.1 Hz), 140.1, 148.8, 161.2 (d, ^1^*J*_CF_ 242.8 Hz), 161.3, 175.3; MS *m/z* (100, MH^+^) 346; HRMS (ES): MH^+^, found 346.1246. C_22_H_17_FNO_2_^+^ requires 346.1243.

## 4. Crystal Structure Solution and Refinement

X-ray quality crystals of the title compound **3f** were obtained by slow crystallization from ethanol solution. Intensity data were collected on a Bruker APEX II CCD area detector diffractometer with graphite monochromated Mo *K*_α_ radiation (50 kV, 30 mA) using the Bruker APEX 2 [30] data collection software. The collection method involved ω-scans of width 0.5° and 512 × 512 bit data frames. Data reduction was carried out using the program Bruker SAINT*+* [31]. The crystal structure was solved by direct methods using Bruker SHELXTL [32]. Non-hydrogen atoms were first refined isotropically followed by anisotropic refinement by full matrix least-squares calculations based on *F*^2^ using *SHELXTL*. Hydrogen atoms were first located in the difference map then positioned geometrically and allowed to ride on their respective parent atoms. Diagrams and publication material were generated using SHELXTL, PLATON [33] and ORTEP-3 [34].

## 5. Conclusions

Overall, the results described in this investigation present another example showing the potential of 2-aryl-4-chloroquinolines in the synthesis of novel 2,3,4-trisubstituted quinolines and the 2,3-diarylquinolin-4(1*H*)-ones with potential to serve as molecular organic materials in nanomaterials or as selective cyclooxygenase-1/-2 (COX-1/-2) inhibitors. Polyarylquinoline–based compounds constitute an important component in optoelectronic materials [24-26]. This moiety constitutes a π-conjugated bridge in nonlinear optical polymers [27] and also serves as electron-acceptor unit in carbazole–quinoline and phenothiazine–quinoline copolymers and oligomers found to exhibit intramolecular charge transfer [28]. The 2,3,4-triarylquinoline derivatives prepared in this investigation can also serve as substrates for metalation with iridium, for example, to form cyclometalated iridium complexes with potential application in organic light-emitting diodes (OLEDs) [25,26] or novel red-emitting electrophosphorescent devices [29]. Studies are currently underway in our laboratory to investigate the biological and photophysical properties of the polysubstuituted quinolones and their quinoline derivatives.

## Figures and Tables

*Reagents* (i) ArB(OH)_2_ (2.5 equiv.), PdCl_2_(PPh_3_)_2_, PCy_3_, K_2_CO_3_, dioxane-water (3:1, v/v); heat, 18 h

**Table:** 

Comp	4-R	Ar	% Yield (3)
**a** **b** **c** **d** **e** **f** **g** **h**	H F Cl OMe H F Cl OMe	-C_6_H_5_ -C_6_H_5_ -C_6_H_5_ -C_6_H_5_ *p*-FC_6_H_4_- *p*-FC_6_H_4_- *p*-FC_6_H_4_- *p*-FC_6_H_4_-	59 55 61 58 72 75 62 62

*Reagents* (i) *p*-FC_6_H_4_B(OH)_2_, Pd(PPh_3_)_4_, 2M K_2_CO_3_, DMF, heat, 48 h; (ii) NH_2_Ph, dioxane, heat, 18 h

**Table:** 

4-R	% Yield (2)	% Yield (4)
H F Cl OMe	60 (**e**) 55 (**f**) 61 (**g**) 79 (**h**)	53 (**a**) 52 (**b**) 69 (**c**) 61 (**d**)

*Reagents*: (i) AcOH-Water (4:1, v/v), heat, 6 h

**Table:** 

Comp	% 4-R	% Yield (5)
**a** **b** **c** **d**	H F Cl OMe	70 70 65 65

**Table 1:** Selected torsion angles (°) for **3f**. For atom labelling see Figure 1.

Ring	Torsion angles/deg (molecule A)		Torsion angles/deg (molecule B)	
2-Ar	N(1)-C(1)-C(22)-C(23)	42.09°	N(2)-C(28)-C(49)-C(50)	60.22°
	C(2)-C(1)-C(22)-C(27)	45.80°	C(29)-C(28)-C(49)-C(54)	60.07°
3-Ar	C(1)-C(2)-C(10)-C(11)	68.03°	C(30)-C(29)-C(37)-C(42)	68.93°
	C(3)-C(2)-C(10)-C(15)	67.27°	C(28)-C(29)-C(37)-C(38)	66.95°
4-Ar	C(2)-C(3)-C(16)-C(17)	68.08°	C(31)-C(30)-C(43)-C(48)	74.75°
	C(4)-C(3)-C(16)-C(21)	68.29°	C(29)-C(30)-C(43)-C(44)	71.34°

**Table 2:** Crystal data and structure refinement for compound **3f**.

Empirical formula Formula weight Temperature Wavelength Crystal system Space group Unit cell dimensions Volume Z Density (calculated) Absorption coefficient F(000) Crystal size Theta range for data collection Index ranges Reflections collected Independent reflections Completeness to theta = 27.00° Absorption correction Max. and min. transmission Refinement method Data / restraints / parameters Goodness-of-fit on F2 Final R indices [I>2sigma(I)] R indices (all data) Largest diff. peak and hole	C_56_H_38_F_6_N_2_O 868.88 173(2) K 0.71073 Å Triclinic P-1 a = 10.2571(2) Å α = 103.2890(10)°. b = 13.2887(2) Å β = 99.4540(10)°. c = 16.7681(3) Å γ = 96.9390(10)°. 2164.00(7) Å3 2 1.333 Mg/m3 0.097 mm−1 900 0.44 × 0.37 × 0.37 mm3 1.27 to 27.00°. -13<=h<=13, -16<=k<=16, -21<=l<=21 40665 9440 [R(int) = 0.0484] 100.0 % None 0.9650 and 0.9586 Full-matrix least-squares on F2 9440 / 0 / 588 1.055 R1 = 0.0424, wR2 = 0.1057 R1 = 0.0640, wR2 = 0.1158 0.218 and -0.379 e.Å−3
